# Severely Dilated Cardiomyopathy and Cardiogenic Shock in a Patient With Thyroid Storm

**DOI:** 10.1210/jcemcr/luaf231

**Published:** 2025-10-24

**Authors:** Mehdia Amini, Jessica Liebich, Guoyu Ling

**Affiliations:** Department of Internal Medicine, Division of Endocrinology, St Louis University, St Louis, MO 63110, USA; Department of Internal Medicine, St Louis University, St Louis, MO 63110, USA; Department of Internal Medicine, Division of Endocrinology, St Louis University, St Louis, MO 63110, USA

**Keywords:** thyroid storm, dilated cardiomyopathy, cardiogenic shock, Graves disease, thyrotoxicosis

## Abstract

Thyroid storm describes a rare, life-threatening hypermetabolic state characterized by an excessive activation of the sympathetic nervous system and heightened sensitivity to circulating catecholamines. We present a case of thyroid storm with severe dilated cardiomyopathy (DCM) and cardiogenic shock.

A 39-year-old woman with a history of hyperthyroidism was admitted following ventricular fibrillation arrest and cardiogenic shock requiring extracorporeal membrane oxygenation (ECMO) support. Clinical findings included proptosis, diffuse thyromegaly, acute kidney injury, shock liver, severe metabolic acidosis, and a severely dilated heart with an ejection fraction of 17%. Thyroid function tests confirmed Graves disease. The patient was treated with methimazole, hydrocortisone, and cholestyramine. Her condition improved, enabling successful weaning from ECMO and vasopressors with recovery of cardiac function. The patient was discharged to rehabilitation on heart failure therapy and methimazole, with follow-up arranged.

This case highlights the rare occurrence of DCM secondary to thyroid storm, underscoring the importance of early recognition and treatment. Thyrotoxicosis can lead to significant cardiac dysfunction, but timely intervention can reverse myocardial damage and improve outcomes. This report emphasizes the need for individualized treatment approaches, especially in cases complicated by coexisting conditions such as hepatic dysfunction.

## Introduction

Thyroid storm is a rare, multisystemic, life-threatening emergency with a reported mortality rate of up to 25% if not promptly treated [1, 2]. Although the precise pathophysiology remains unclear, it is typically triggered by an acute physiological stressor in individuals with underlying thyrotoxicosis [3]. The excessive activation of the sympathetic nervous system and heightened sensitivity to catecholamines results in profound systemic effects, including cardiovascular, thermoregulatory, and metabolic disturbances, which can rapidly progress to multiorgan dysfunction if left untreated [4]. The cardiovascular system is particularly vulnerable, with thyroid storm known to precipitate tachyarrhythmias, high-output heart failure and, in severe cases, cardiogenic shock [5]. Early recognition is crucial as timely intervention and management can reverse the myocardial damage and improve mortality.

We present the case of a 39-year-old patient with thyroid storm complicated by cardiogenic shock and severe dilated cardiomyopathy (DCM), an exceedingly rare manifestation of thyroid storm.

## Case Presentation

A 39-year-old woman with a medical history of hyperthyroidism and asthma was admitted following a witnessed ventricular fibrillation arrest. Return of spontaneous circulation was achieved in the field through cardiopulmonary resuscitation and defibrillation. On admission, she was unresponsive, intubated, and in cardiogenic shock with a heart rate of 120 beats per minute and a fever of 102.6 °F (∼39.2 °C). Physical examination revealed proptosis, thyromegaly, elevated jugular venous pressure, bilateral crackles on lung auscultation, and peripheral edema. Due to progressive circulatory collapse, she required extracorporeal membrane oxygenation (ECMO) for hemodynamic support.

The endocrinology team was consulted for concern of thyroid storm. According to the patient's family, she had previously been diagnosed with Graves disease but was nonadherent to medical therapy. The history was limited as the patient lived by herself. In the weeks leading up to her admission, she had experienced insomnia, heat intolerance, diarrhea, weight loss, and proptosis. More recently, she developed shortness of breath, orthopnea, and lower-extremity edema. She had no prior history of cardiac disease or arrhythmia. Her mental status was reported to be intact at baseline.

## Diagnostic Assessment

**Figure 1.**
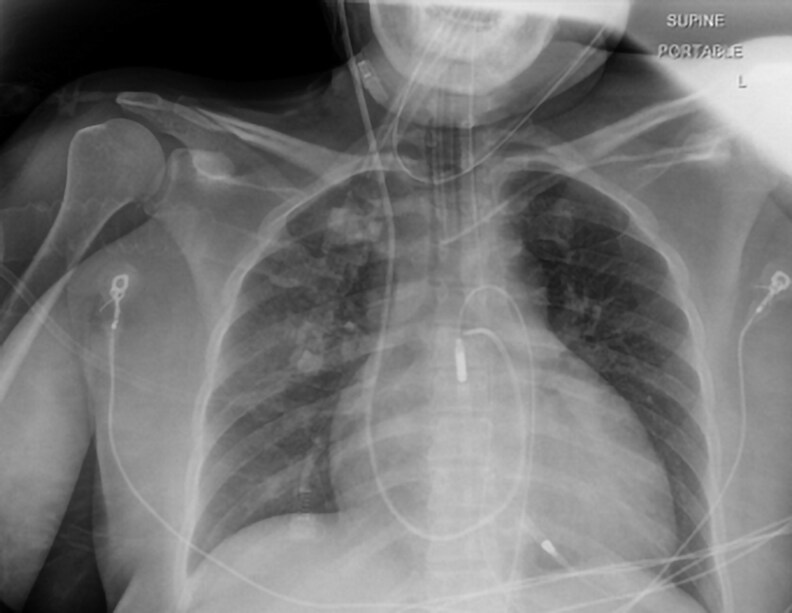
Initial chest radiograph demonstrating cardiomegaly and pulmonary vascular congestion.

**Figure 2.**
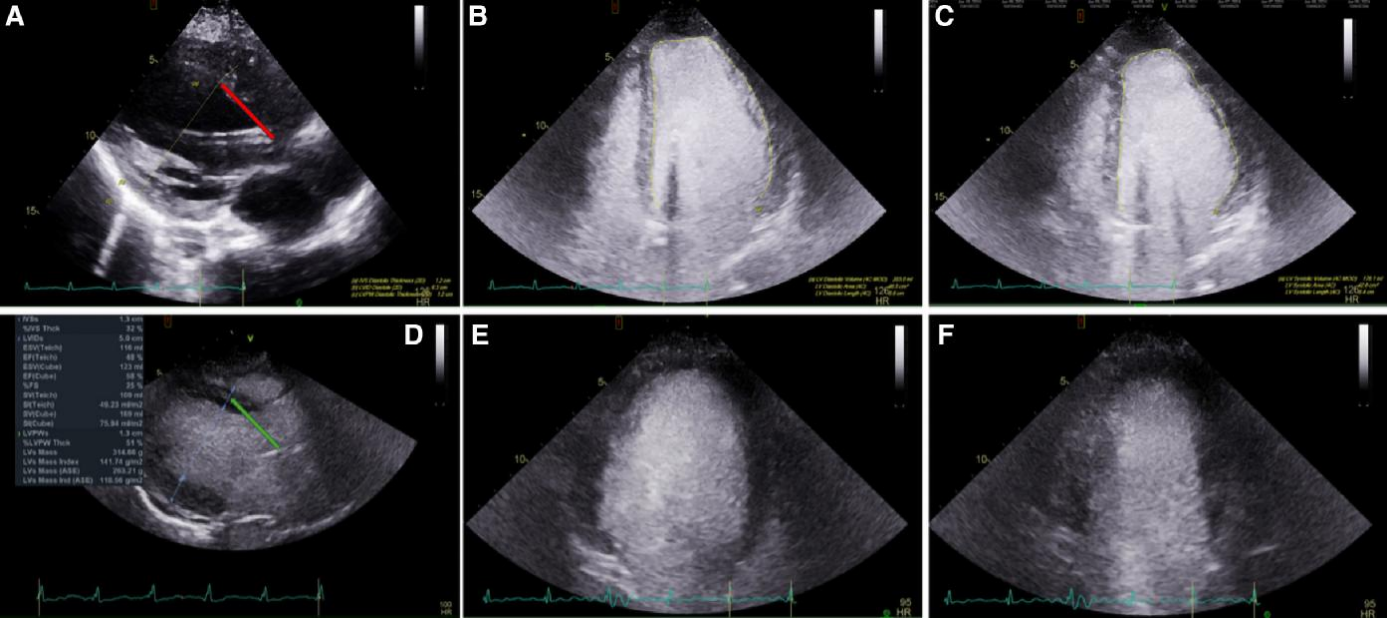
Transthoracic echocardiogram A to C, before and D to F, 2 weeks after thyroid storm treatment. A and D show changes in left ventricular (LV) inner diameter from 6.3 cm initially (A, red line) to 5.0 cm after treatment (D, green line). B and C show the initial contrast-filled LV between B, diastole and C, systole, demonstrating an ejection fraction (EF) of 15% to 20%. E and F show the posttreatment contrast-filled LV between E, diastole and F, systole, demonstrating an EF of 48%.

**Figure 3.**
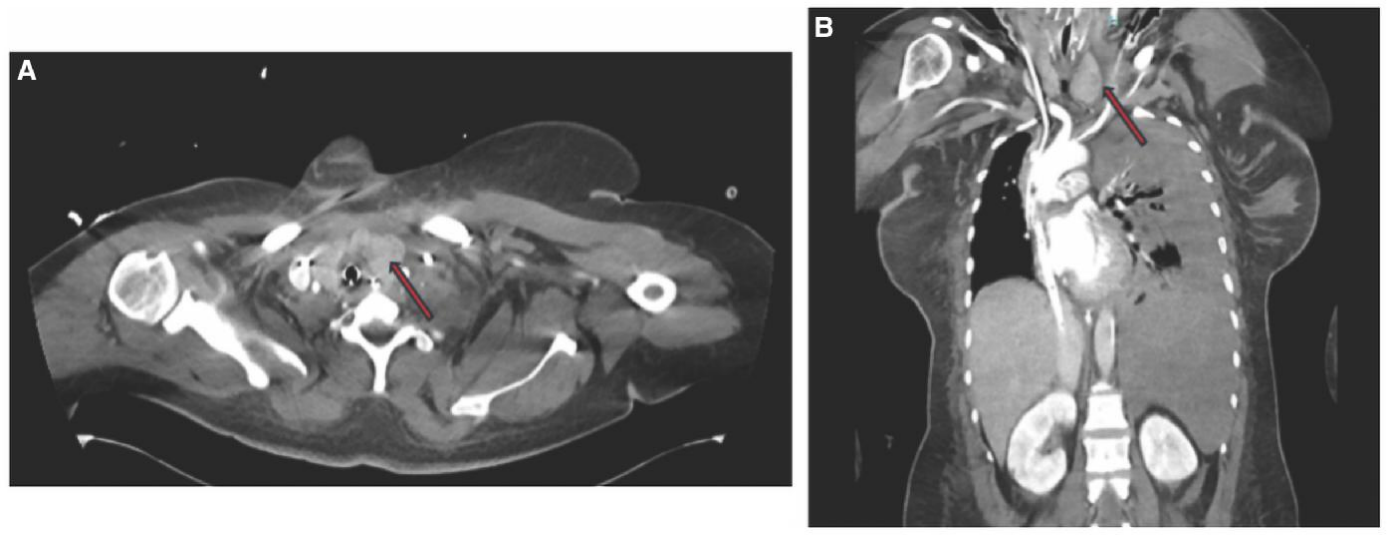
Computed tomography chest A, axial and B, coronal view, demonstrating an asymmetrically enlarged left thyroid lobe.

Her initial laboratory work-up revealed an acute kidney injury with a creatinine of 2.29 mg/dL (202.3 µmol/L) (normal reference range [RR], 0.74-1.35 mg/dL; 65.4-119.3 µmol/L), shock liver with alanine transaminase of 659 U/L (11 µkat/L) (normal RR, 7-56 U/L; 0.12-0.93 µkat/L), aspartate transaminase of 2140 U/L (35.7 µkat/L) (normal RR, 10-40 U/L; 0.17-0.67 µkat/L), and severe metabolic acidosis with a lactic acid of 12.5 mmol/L (normal RR, 0.5-2.0 mmol/L). The initial electrocardiogram showed sinus tachycardia and nonspecific T-wave inversion. Her chest x-ray showed cardiomegaly and pulmonary vascular congestion (Fig. 1). A transthoracic echocardiogram (TTE) showed severe cardiac dilatation with concentric hypertrophy and an ejection fraction (EF) of 15% to 20% (normal RR, 55%-70%) (Fig. 2A-2C). She was found to have an asymmetrically enlarged left thyroid lobe on computed tomography (CT) scan (Fig. 3). Further evaluation revealed a suppressed thyroid stimulating hormone (TSH) (<0.01 µIU/mL) (normal RR, 0.4-4.0 µIU/mL) with modestly elevated free thyroxine (FT4) 2.6 ng/dL) (33.4 pmol/L) (normal RR, 0.8-1.8 ng/dL; 10.3-23.2 pmol/L), and a markedly elevated free triiodothyronine (FT3) of 13.9 pg/mL (1.7-3.7 pg/mL). The thyroid-stimulating immunoglobulin level was markedly elevated at 3.46 IU/L (normal RR <1.75 IU/L), consistent with Graves disease. Baseline complete blood count and absolute neutrophil count were normal.

## Treatment

The patient was initiated on methimazole 20 mg every 6 hours, intravenous hydrocortisone 100 mg every 8 hours, and cholestyramine 4 g every 6 hours. β-Blockers were not initiated as they are contraindicated in cardiogenic shock due to their negative inotropic and chronotropic effects, which can further impair cardiac output and exacerbate hemodynamic instability. Her liver function was closely monitored during methimazole therapy due to the risk of hepatotoxicity.

## Outcome and Follow-up

**Figure 4.**
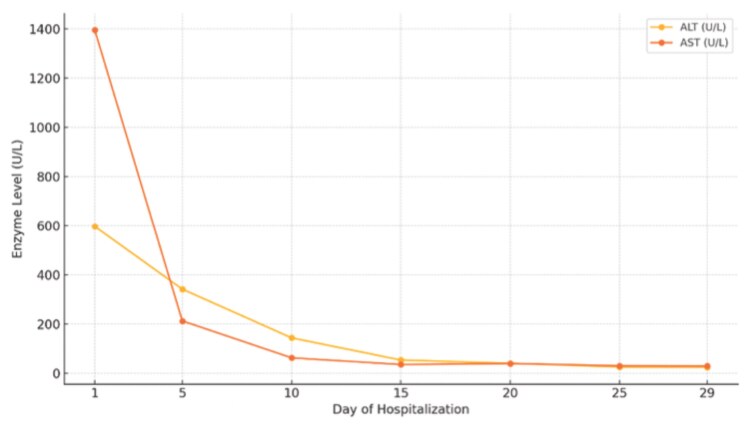
Trend of liver function tests during hospitalization.

Over the following days, the patient showed significant clinical improvement. ECMO and vasopressor support were successfully weaned, and she was extubated. Her fever resolved, and infectious work-up was negative. Laboratory markers, including FT4, total T3, liver enzymes, and creatinine, progressively normalized (Table 1 and Fig. 4). As her condition stabilized, cholestyramine was discontinued, steroids were tapered, and methimazole was reduced to 5 mg daily. A repeat TTE showed an improvement in left ventricular (LV) inner diameter from 6.3 cm to 5.0 cm and EF from 15% to 20% to 48% (Fig. 2). The patient was discharged to an acute rehabilitation facility on goal-directed medical therapy for heart failure. She was continued on methimazole 5 mg daily followed by a total thyroidectomy a few months later. At the time of discharge, the patient exhibited signs of encephalopathy, including cognitive slowing and forgetfulness. However, on follow-up, these symptoms had fully resolved.

**Table 1.** Trend of thyroid functions from admission to discharge

Thyroid function test	Day 1	Day 9	Day 13	Day 19	Day 28	Reference range
TSH	**<0.010 μIU/mL**	**<0.010 μIU/mL**	**<0.010 μIU/mL**	**<0.010 μIU/mL**	0.037 μIU/mL	0.350-4.940 μIU/mL
Free T4	**2.6 ng/dL (33.5 pmol/L)**	0.9 ng/dL (11.6 pmol/L)	**0.6 ng/dL (7.7 pmol/L)**	0.7 ng/dL (9.0 pmol/L)	0.7 ng/dL (9.0 pmol/L)	0.7-1.5 ng/dL (9.0-19.3 pmol/L)

Abnormal values are shown in bold. Values in parentheses are in International System of Units (SI).

Abbreviations: FT4, free thyroxine; TSH, thyroid stimulating hormone.

## Discussion

This case highlights the rare but serious cardiovascular complications of thyroid storm, particularly thyrotoxicosis-induced DCM. Thyrotoxicosis exerts profound effects on cardiac function through T3-mediated mechanisms. T3 binds to intracellular thyroid hormone receptors and modulates the expression of genes involved in calcium cycling, which are critical for myocardial contraction and relaxation [6]. T3 also increases the depolarization and repolarization rates of the sinoatrial node and shortens the refractory period of cardiac myocytes, predisposing patients to tachyarrhythmias [7]. The increased density of β-adrenergic receptors on atrial myocytes further raises susceptibility to atrial fibrillation and supraventricular tachycardia in hyperthyroid states. Additionally, T3 enhances β-adrenergic receptor sensitivity to catecholamines, contributing to tachyarrhythmias and hemodynamic instability [8, 9]. Furthermore, thyroid hormone reduces systemic vascular resistance through arterial smooth muscle relaxation, which triggers compensatory activation of the renin-angiotensin-aldosterone system and increases sodium and water retention [10]. T3 also stimulates erythropoietin production, augmenting circulating blood volume by up to 25% and increasing cardiac preload and output. Collectively, these effects lead to high-output heart failure, driven by increased inotropy, chronotropy, and elevated circulating volume. Thyrotoxicosis can increase cardiac output by up to 300%, resulting in significant hemodynamic burden [11].

On presentation, our patient had more pronounced elevated FT3, 3.7-fold over the upper limit of normal, whereas her FT4 was 1.7-fold over the upper limit of normal. Whether disproportionately elevated FT3 compared to FT4 elevation is a risk of adverse cardiovascular events warrants further research.

DCM secondary to thyrotoxicosis is an extremely rare but serious complication of thyroid storm, with an incidence of less than 1% of all patients with thyrotoxicosis-induced cardiomyopathy [5]. The pathophysiology is likely multifactorial and includes direct thyroid hormone-induced myocardial toxicity, altered myocyte energy metabolism, and sustained tachycardia-induced myocardial strain. While older patients with preexisting cardiovascular conditions such as hypertension, ischemic heart disease, or valvular pathology are at a greater risk, thyrotoxicosis-induced DCM has also been described in younger patients without comorbidities [5, 6]. Untreated DCM can lead to irreversible cardiac remodeling, reduced EF, persistent arrhythmias, and cardiogenic shock. However, when identified early, thyrotoxicosis-induced DCM is potentially reversible with early intervention, distinguishing it from other forms of DCM that often involve irreversible remodeling [12]. This is also demonstrated by a rapid improvement in the echocardiogram findings, including a lesser dilated LV from 6.3 to 5 cm, and improvement of LV EF from 15% to 20% to 48%, just 2 weeks after aggressive medical management (Fig. 2A-2C vs 2D-2F).

A unique challenge in this case was managing thyroid storm in the context of abnormal liver function tests (LFTs). Although LFT abnormalities can complicate antithyroid drug (ATD) therapy, there are no specific guidelines for initiating ATDs in patients with an acute liver injury from other causes. In our patient, methimazole was started with careful monitoring of LFTs, in accordance with American Thyroid Association guidance favoring individualized treatment plans in complex cases [13]. However, the use of ATDs in acute liver injury remains controversial, with some reports advocating therapeutic plasma exchange as an alternative treatment modality [14]. These ongoing debates emphasize the need for more robust guidelines in the management of thyroid storm with hepatic dysfunction.

Neurologic recovery is another important aspect of thyroid storm management. While our patient's hemodynamics improved promptly, her cognitive recovery lagged. The literature suggests that altered mental status is often reversible, but recovery timelines vary depending on metabolic derangements and the severity and duration of thyrotoxicosis [2]. In this case, a follow-up visit after rehabilitation, approximately 2 months from thyroid storm, documented a normal mental status examination, indicating a complete recovery of her encephalopathy.

The use of vasopressors during cardiogenic shock presents a paradoxical challenge in thyroid storm. Though necessary for hemodynamic support in cardiogenic shock, vasopressors can amplify the underlying hyperadrenergic state inherent in thyroid storm, potentially exacerbating the crisis.

The limitations of this case include the inability to definitively determine the sequence of events leading to thyroid storm and DCM. Although our patient was diagnosed with hyperthyroidism prior to her presentation, DCM has been reported as the initial manifestation of thyrotoxicosis in some cases [15], not necessarily leading to acute decompensated organ failures. The chronology of events may not always be clear.

We propose that long-standing hyperthyroidism led to DCM and subsequent acute decompensation. The acute catecholamine surge from the resulting ventricular fibrillation may have intensified sympathetic drive, precipitating thyroid storm, which then in turn exacerbates organ failure. Notably, vasopressors used for cardiogenic shock may have also worsened the thyroid storm by intensifying the hyperadrenergic state, a limitation that complicates therapeutic decisions.

Finally, while individual case reports and small series provide valuable insight into rare presentations, larger studies are needed to guide optimal management strategies, particularly in complex presentations involving coexisting hepatic dysfunction or delayed mental status recovery. This case underscores the importance of early diagnosis, aggressive treatment, and individualized care in managing thyroid storm and its complications, as timely intervention can substantially improve outcomes and prevent irreversible sequelae.

## Learning Points

- Thyroid storm, a life-threatening condition, can lead to severe cardiovascular complications, including arrhythmias, cardiogenic shock, and heart failure, even in patients without prior cardiac disease.
- Thyrotoxicosis-induced DCM is extremely rare but reversible with early recognition and appropriate treatment, highlighting the need for prompt management of thyroid storm.
- β-Blockers, often essential for managing thyroid storm, may be contraindicated in cases of cardiogenic shock, necessitating individualized treatment strategies with alternatives like ECMO.
- Methimazole, though effective, carries a risk of hepatotoxicity, particularly in patients with preexisting liver dysfunction, requiring careful monitoring throughout treatment, especially in patients with acute liver injury.
- Successful outcomes in thyroid storm cases often require coordinated care between endocrinology, cardiology, and critical care teams to manage both endocrine and cardiovascular complications.

## Data Availability

Original data generated and analyzed during this study are included in this published article.

## Abbreviations

ATD: antithyroid drug
CT: computed tomography
DCM: dilated cardiomyopathy
ECMO: extracorporeal membrane oxygenation
EF: ejection fraction
FT3: free triiodothyronine
FT4: free thyroxine
LFTs: liver function tests
LV: left ventricular
RR: reference range
TSH: thyroid stimulating hormone
TTE: transthoracic echocardiogram
